# Supplementary material for: Ankyrin-G and Its Binding Partners in Neurons: Orchestrating the Molecular Structure of the Axon Initial Segment
Source: Biomolecules. 2025 Jun 19;15(6):901. doi: 10.3390/biom15060901 (PMC12190637; doi:10.3390/biom15060901)
Supplement: Supplementary file 1 [file biomolecules-15-00901-s001.zip › biomolecules-3672417-supplementary.pdf]

Supplementary Table S1 Seventy-one high-confidence AIS proteins and AIS localization analysis results by immunohistochemistry.

Ranking	**Protein names (known AIS proteins: shadow)**	**Gene names**	**AIS Localization By immunohistochemistry**	**References**
1	Tripartite motif-containing protein 46	*Trim46*	++	[24]
2	Spectrin beta chain, non-erythrocytic 4 isoform sigma6	*Sptbn4*	++	[25]
3	Ankyrin-3 (Ankyrin-G)	*Ank3*	++	[17]
4	Sodium channel protein type 2 subunit alpha (Voltage-gated sodium channel subunit alpha Nav1.2)	*Scn2a*	++	[17]
5	Neurofascin	*Nfasc*	+	[26]
6	WD repeat-containing protein 7 (TGF-beta resistance-associated protein TRAG)	*Wdr7*	+	[19]
7	Tenascin-R (TN-R)	*Tnr*	+	[27]
8	Scribble planar cell polarity protein	*Scrib*	+	[19]
9	WD repeat domain 47	*Wdr47*	+	[19]
10	Zinc finger ZZ-type and EF-hand domain-containing 1	*Zzef1*	n/a	
11	F-box protein 41	*Fbxo41*	n/a	
12	Protein fem-1 homolog B (FEM1b)	*Fem1b*	n/a	
13	2-lysophosphatidate phosphatase PLPPR4	*Plppr4 Lppr4*	n/a	
14	NAD (+) hydrolase SARM1 (NADase SARM1)	*Sarm1*	n/a	
15	Transmembrane anterior-posterior transformation 1	*Tapt1*	n/a	
16	Band 4.1 (Erythrocyte membrane protein band 4.1)	*Epb41 Epb4.1*	para-AIS	[28]
17	Eukaryotic translation initiation factor 3 subunit I (eIF3i)	*Eif3i Eif3s2*	n/a	
18	Versican core protein (Chondroitin sulfate proteoglycan core protein 2)	*Vcan Cspg2*	+	[29]
19	FERM, ARHGEF, and pleckstrin domain-containing protein 1 (FERM, RhoGEF, and pleckstrin domain-containing protein 1)	*Farp1*	n/a	
20	Lissencephaly-1 protein (LIS-1)	*Lis-1*	+	[30]
21	Echinoderm microtubule-associated protein (EMAP)-like 4	*Eml4*	n/a	
22	Vacuolar protein sorting-associated protein 41 homolog	*Vps41*	n/a	
23	Voltage-dependent P/Q-type calcium channel subunit alpha-1A	*Cacna1a*	Only physiologically certified	[31]
24	EMAP-2	*Eml2 Emap2*	n/a	
25	D-3-phosphoglycerate dehydrogenase	*Phgdh*	n/a	
26	Xaa-Pro aminopeptidase 3 (X-Pro aminopeptidase 3)	*Xpnpep3*	n/a	

27	Golgi brefeldin A-resistant guanine nucleotide exchange factor 1	*Gbf1*	n/a	
28	Geranylgeranyl transferase type-2 subunit alpha	*Rabggta Ggta*	n/a	
29	Brevican core protein	*Bcan Behab*	+	[26]
30	AMP deaminase 3 (EC 3.5.4.6) (AMP deaminase isoform E)	*Ampd3*	n/a	
31	SET-binding factor 2	*Sbf2*	n/a	
32	Synaptotagmin-11	*Syt11*	n/a	
33	Plasma membrane calcium-transporting ATPase 3	*Atp2b3 Pmca3*	n/a	
34	Sodium channel subunit beta-2	*Scn2b*	Other isoforms express	[32,33]
35	Baculoviral IAP repeat-containing 6	*Birc6*	n/a	
36	ATP-binding cassette sub-family D member 3 (70 kDa peroxisomal membrane protein) (PMP70)	*Abcd3 Pmp70*	n/a	
37	Autophagy-related protein 9A (APG9-like 1)	*Atg9a Apg9l1*	n/a	
38	Glutamate decarboxylase 2 (GAD-65)	*Gad2 Gad65*	+	[34]
39	AP-2 complex subunit alpha-2	*Ap2a2 Adtab*	n/a	
40	RB1-inducible coiled-coil 1	*Rb1cc1*	n/a	
41	RAN-binding protein 6	*Ranbp6*	n/a	
42	WD repeat and FYVE domain-containing 3	*Wdfy3*	n/a	
43	RCR-type E3 ubiquitin transferase	*Mycbp2*	n/a	
44	Sodium/potassium-transporting ATPase subunit alpha-2 (Na (+)/K (+) ATPase alpha-2 subunit)	*Atp1a2*	n/a	
45	Epidermal growth factor receptor pathway substrate 15	*Eps15*	n/a	
46	Fatty acid synthase	*Fasn*	n/a	
47	Sodium/potassium-transporting ATPase subunit alpha-3	*Atp1a3*	n/a	
48	Adenylate cyclase 9 (Adenylate cyclase 9 (Predicted), isoform CRA_b)	*Adcy9*	n/a	
49	Gamma-tubulin complex component	*Tubgcp3*	n/a	
50	Sideroflexin-5 (Tricarboxylate carrier BBG-TCC)	*Sfxn5*	n/a	
51	Disco-interacting protein 2 homolog B	*Dip2b*	n/a	
52	Nck-associated protein 1 (NAP 1) (Membrane-associated protein HEM-2) (p125Nap1)	*Nap1*	n/a	
53	NBAS subunit of the NRZ tethering complex	*Nbas*	n/a	
54	Peroxiredoxin-5, mitochondrial	*Prdx5*	n/a	
55	Fibronectin (FN) [Cleaved into: Anastellin]	*Fn1*	n/a	
56	Protein Rogdi homolog	*Rogdi*	n/a	
57	Ankyrin repeat and FYVE domain-containing 1	*Ankfy1*	n/a	
58	Neurofibromin (Neurofibromatosis-related protein NF-1)	*Nf1*	n/a	
59	Disco-interacting protein 2 homolog A	*Dip2a*	n/a	
60	Cytoskeleton-associated protein 2	*Ckap2*	n/a	
61	Peroxisomal biogenesis factor 11 beta (Peroxisomal biogenesis factor 11b, isoform CRA_b)	*Pex11b*	n/a	
62	Integrin-linked protein kinase	*Ilk*	n/a	
63	Ribosomal protein S27-like	*LOC100362987*	n/a	

64	Cullin-7 (CUL-7)	*Cul7*	n/a
65	F-box/LRR-repeat protein 16 (F-box and leucine-rich repeat protein 16)	*Fbxl16 Scirr1*	n/a
66	Echinoderm microtubule-associated protein-like 1 (EMAP-1)	*Eml1*	n/a
67	Microtubule-associated protein 1S (MAP-1S)	*Map1s*	n/a
68	Glutathione S-transferase theta-2	*Gstt2*	n/a
69	AP-2 complex subunit sigma (Adaptor protein complex AP-2 subunit sigma)	*Ap2s1*	n/a
70	Ankyrin repeat domain 28	*Ankrd28*	n/a
71	S-adenosylhomocysteine hydrolase-like protein 1 (IP3R-binding protein released with inositol 1,4,5-trisphosphate)	*Ahcyl1 Irbit*	n/a
